# Fascinating or dull? Female students’ attitudes towards STEM subjects and careers

**DOI:** 10.3389/fpsyg.2022.959972

**Published:** 2022-09-29

**Authors:** Ciara Lane, Sila Kaya-Capocci, Regina Kelly, Tracey O’Connell, Merrilyn Goos

**Affiliations:** ^1^EPI*STEM, National Centre for STEM Education, School of Education, University of Limerick, Limerick, Ireland; ^2^Faculty of Education, Agri Ibrahim Cecen University, Agri, Turkey; ^3^School of Education, University of Limerick, Limerick, Ireland

**Keywords:** STEM, attitude, gender, awareness, ability, value, commitment

## Abstract

Internationally, the need to advance science, technology, engineering and mathematics (STEM) education is recognized as being vital for meeting social and economic challenges and developing a scientifically, mathematically, and technologically literate citizenry. In many countries, however, there are gender differences in the participation and achievement of girls and women in STEM education and STEM careers, usually to the disadvantage of females. This paper aims to identify challenges to female students’ participation in STEM both at post-primary (secondary school) level and beyond in the Irish context. The research questions we aim to address in this paper are: (1) what are student attitudes towards science, technology, engineering and mathematics as measured through interest and perceived ability in STEM, students’ valuing of STEM and students’ commitment to STEM? and (2) what gender differences occur regarding students’ attitudes to science, technology, engineering and mathematics? A survey was completed by 308 post-primary students in Ireland as part of a one-year research project titled “STEMChAT: Women as catalysts for change in STEM education.” Data analysis compiled descriptive statistics, including response frequencies and percentages and median and interquartile range values, and compared gender differences in survey responses using the Kruskal–Wallis H Test. Results indicated that female students had significantly more positive attitudes to science compared to males while in comparison, males had significantly more positive responses to mathematics compared to females. Challenges regarding access to and understanding of STEM in the context of post-primary education are discussed.

## Introduction

Internationally, there has been an increased emphasis on science, technology, engineering and mathematics (STEM) education due to its significant impact on social, environmental and/or economic development (Kelley and Knowles, 2016; Martin-Paez et al., 2019). Even though STEM education is a highly studied topic, there are still disputes about its meaning. While some researchers explain STEM education with a simple description of the four STEM disciplines (science, technology, engineering and mathematics), others view it as an educational approach at the intersections of any number of the four disciplines. For example, different researchers view STEM education as:

- Science or Maths,
- both Science and Maths,
- Science by incorporating Technology,
- Engineering or Maths,
- a quartet of separate STEM disciplines,
- Science and Maths are connected by a Technology or Engineering program,
- coordination across STEM disciplines,
- combining two or three STEM disciplines,
- complementary overlapping across STEM disciplines,
- transdisciplinary STEM course or program.

Bybee (2013)

Additionally, some studies adopting a more complex understanding view STEM education as merging all four STEM disciplines in an integrated manner (McLoughlin et al., 2020). This study was conducted in Ireland, where the meaning of STEM education is multi-faceted and can include the teaching of the four STEM disciplines in isolation as well as encouragement for cross-disciplinary approaches, especially in primary schools (Department of Education and Skills [DES], 2017). However, subjects taught in Irish post-primary schools are not integrated; rather, students study subjects from the constituent STEM disciplines in a discrete manner.

In many countries it is recognised that providing effective STEM education at primary, secondary and tertiary levels is vital to increase the number and quality of STEM graduates (Marginson et al., 2013; Honey et al., 2014; DES, 2017; The Scottish Government, 2017). In an increasingly global society, it is important for all students to develop ‘STEM literacy’ to meet social, personal, economic and environmental challenges (Mohr-Schroeder et al., 2020) and, thus, STEM education has come to the fore of national and global policies in recent decades. However, in Ireland, as in many other countries worldwide, gender differences in the participation and achievement of girls and women in STEM education and the STEM workforce are palpable, usually to the disadvantage of females.

In 2018, Ireland had the highest rate of STEM graduates in the EU at 35.2 per 1,000 persons aged 20–29 (Central Statistics Office, 2021). However, Ireland also had the largest gender differential in the EU, with 47.3 male STEM graduates per 1,000 compared to 23.0 female STEM graduates. The gender gap problem is often portrayed as a “leaky pipeline,” with low female participation in second-level STEM subjects leading to similarly low participation rates in third-level STEM programs. In Ireland, there are many informal activities available to students, both male and female, that are designed to boost participation in STEM education and STEM careers. These include the BT Young Scientist Awards (BT Young Scientist and Technology Exhibition, 2021), CoderDojo (CoderDojo Foundation, n.d.), SciFest (2021), and RDS STEM Learning (RDS, 2021). However, the benefits of these informal initiatives are not fully realised unless education systems and schools also provide equal opportunities for boys and girls to access and benefit from quality STEM education.

This paper draws on survey data collected as part of a Science Foundation Ireland (SFI) funded research project titled “STEMChAT: Women as catalysts for change in STEM education” aiming to encourage female post-primary students to pursue STEM subjects and careers. The survey was conducted with post-primary students from 12 schools before STEMChAT activities were introduced (school-based workshops involving conversations with female undergraduate STEM students about university courses and careers). As such, findings reflect participants’ pre-existing attitudes to STEM. The conceptual framework for our study is informed by the UNESCO (2017) Ecological Framework, which depicts the multiple and overlapping factors that may influence girls’ and women’s participation, achievement and progression in STEM studies and careers. These factors are described at four interactive levels: individual; family and peer; school; and society. At the individual level, cognitive traits such as spatial or linguistic skills may be influential, along with psychological factors that include self-efficacy, interest and motivation. Family and peer-related factors highlight the role of parental beliefs, expectations, educational and occupational backgrounds; the household environment and resources; and peer relationships. School-level factors include the learning environment, teacher characteristics, teaching strategies, the curriculum and learning materials, and assessment procedures and tasks. Societal and cultural norms can reinforce or challenge gender stereotypes, and at this societal level of the Ecological Framework the mass media and social media are significant influences on the socialisation of children and young people. Also, at the societal level, formal policies and legislation can also promote gender equality and the advancement of women in the STEM fields.

While it is difficult to investigate the Ecological Framework’s multiple levels and inter-related factors in a single study, attention paid to any influential factor or level (e.g., the psychological factors at the individual level) must also take into account the interactions of the other levels and factors (e.g., family, peer, school and societal factors). Although this paper focuses on specific psychological factors at the individual level (students’ attitudes), we are cognizant of the various other factors at other levels in which our study is contextualized. The research questions we aim to address in this paper are:

1. What are student attitudes towards science, technology, engineering and mathematics as measured through:
  - awareness of STEM (initial interest)
  - perceived ability in STEM
  - students’ valuing of STEM
  - students’ commitment to STEM (long-term interest)?
2. What gender differences occur regarding students’ attitudes to science, technology, engineering and mathematics?

Our paper firstly introduces STEM education in the Irish context and then discusses relevant literature with regard to students’ attitudes to STEM, including gender differences. A quantitative analysis is conducted on the survey data collected from post-primary students. The results are discussed in terms of access to STEM subjects and students’ attitudes and gender differences, and the limitations of the study are presented. The paper concludes with the contributions to the STEM education field by identifying the challenges to female students’ participation in STEM and providing potential research areas to address these challenges.

## STEM education in Ireland

The *STEM Education Policy Statement 2017–2026* released by the Department of Education and Skills (2017) in Ireland reveals a vision for providing “the highest quality STEM education experience for learners that nurtures curiosity, inquiry, problem-solving, creativity, ethical behaviour, confidence and persistence, along with the excitement of collaborative innovation” (p. 12). The policy statement underlines the built-in educational benefits of inspiring young people’s curiosity about the natural world while also highlighting the importance of producing STEM graduates to drive Ireland’s economy. The policy statement discusses the necessity of high-quality STEM education for all students, not only those interested in STEM-related careers, due to its importance in creating STEM-literate citizens who can make well-informed decisions about global issues affecting future generations. According to the *STEM Education Policy Statement,* engagement and participation of learners with STEM is the first of four pillars of the STEM policy plan for advancement. Therefore, it is highly important to investigate and address problems of low participation in STEM disciplines in Irish post-primary schools.

In Ireland, post-primary education comprises Junior Cycle (the first 3 years) and Senior Cycle (the final 2 years), with an optional Transition Year between Junior and Senior Cycle. At the end of Senior Cycle, students sit the Leaving Certificate examination, the results of which determine their entry to third level courses and careers. Students typically study a minimum of six subjects for Senior Cycle, with English, Irish and mathematics taken by the majority of students, due to these being effectively compulsory (being required for entry to most third level courses). Apart from mathematics, other STEM subject choices for Senior Cycle include applied mathematics, biology, chemistry, physics, physics & chemistry, agricultural science, construction studies, design & communication graphics, and engineering.

Students’ subject choice at Senior Cycle is naturally affected by subjects they completed at Junior Cycle, subjects offered in the school, availability of teachers, and the students’ attitudes towards these subjects, among others. In Ireland, there are dramatic gender imbalances at the post-primary level in favour of male students in physical science and technology subjects and in favour of female students in biology. DES (2017) shows the significant gender differences in the selection of science subjects at Senior Cycle, with the ratios of male students to female students greater than 3:1 for physics and approximately 2:3 for biology. Moreover, the number of female students is considerably lower than male students in STEM courses in higher education.

Access to subjects for female students at post-primary level may be affecting interest and opportunity to study these subjects. Access to subjects in post-primary schools can be difficult at times depending on school budgets and resources in subjects such as engineering or construction studies where tools and space may not be available to equip a practical workshop classroom. In 2019, 325 females sat the Leaving Certificate engineering examination while 4,440 males sat the same exam. Attending single-sex schools, particularly single-sex girls’ schools, affects students’ access to subjects. Many all-girls post-primary schools would not have workshops for practical subjects like construction studies. Single-sex education is common in Irish schools: in 2017, the *Irish Times* newspaper reported that one-third of schools in Ireland catered for either girls only or boys only (Ahlstrom, 2017). This is a structural feature of the education landscape that can reinforce negative gender stereotypes, such as the perception that STEM subjects and careers are more suitable for boys (report by Accenture, 2014).

Gender disparities in STEM education in Ireland are compounded by subject hierarchies and sub-cultures that exist within the post-primary school curriculum. McGarr and Lynch (2017), in their analysis of the STEM agenda in the Irish education system, highlighted the hierarchical ordering of subjects that often reflects the social and educational capital available to those who choose these subjects. The pursuit and performance of students in technology and engineering subjects versus that in mathematics and science subjects is seen as reflective of student ability in these subjects. STEM subjects have generally been presented as one interrelated entity; however, they play different roles in Irish post-primary schools. The vocationally focused subjects of technology and engineering have traditionally served the needs of lower socio-economic groups, while mathematics and science have been viewed as higher-status subjects that prepare middle-class students for university degrees and more privileged occupations. McGarr and Lynch highlighted the absence of technology and engineering from the broader STEM agenda in post-primary schools, which they claimed is largely due to the under-resourced and out-of-date subjects on offer. For example, the curriculum for engineering has been in place without change or update for over two decades. These researchers argued that the academically oriented subjects of science and mathematics monopolize the STEM agenda, while the traditional role of the vocational subjects in preparing students for post-school employment is now downplayed due to the massification of higher education.

Further research on issues in STEM education internationally is discussed in the next section, particularly in relation to attitudes and gender differences.

## Issues in STEM education

Many issues relating to STEM education are raised by international researchers, some of which involve students’ attitudes towards STEM education, particularly regarding gender differences at high school (post-primary) level (Brown et al., 2017). To limit our review of the literature, attitudes towards STEM are characterized as STEM interest, STEM values, and STEM perceived ability according to Mahoney’s (2010) Students’ Attitudes Towards STEM Survey as used in our study, and gender differences are specified as STEM stereotypes. It is acknowledged that these constructs overlap in many research studies, as the following brief review demonstrates.

### Students’ attitudes towards STEM

In the last decade, several studies investigated particular variables that drive or limit interest in STEM (Valla and Ceci, 2014; Falk et al., 2017; Means et al., 2021). In general, females have been found to have lower interest levels in STEM compared to males (Falk et al., 2017). Social inclusion factors are noted as a particular reason for lower interest. Means et al. (2021) reported on a large-scale meta-analysis of the relationship between attendance at an inclusive STEM high school and a range of academic and motivational outcomes. Rather than selecting students on the basis of prior academic achievement, inclusive STEM high schools provide opportunities for under-represented youth to develop STEM interest and talent. The meta-analysis found that students who attended an inclusive STEM high school were more likely than students in non-STEM comparison schools to report high interest in undertaking a graduate degree and in entering a STEM career, and this effect was also found for low-income and female students. Turner et al. (2019) reported on the importance of efficacy in relation to STEM interest; peer support was noted as a contributing factor to efficacy. These studies call on the need to focus on equity-oriented interventions that increase the social belongingness of students in STEM domains where there is unequal participation by gender in order to increase interest in STEM.

Value beliefs have gained increasing attention in the psychology domain in recent years, with value-related beliefs noted as a strong predictor of career aspirations in STEM (Wang et al., 2013; Wegemer and Eccles, 2019). Van Tuijl and Van der Molen (2016) conducted a review of literature regarding the study choice factors that are most influential on children from the age of 8–16. They conclude that the undesirable affective value associated with many STEM fields is detrimental to the career aspirations of young people, particularly those who do not align with the stereotypical image of STEM careers. Tzu-Ling (2019) used multiple regression to investigate the difference between males’ and females’ career aspirations using the variables of task value, self-efficacy and family support. Tzu-Ling reported that task value is a variable that can significantly predict STEM career aspirations, regardless of gender, whereas self-efficacy could significantly predict STEM career aspiration for male students only. Eccles and Wigfield (2020) argued that more research is needed to explore how complex interactions between culture, gender, and ethnicity influence the development of individuals’ subjective task values. These studies highlight the need to develop the cultural and affective value of STEM and STEM tasks as a means to counter the negative values associated with some STEM fields.

Historically, researchers report that females’ lack of interest in STEM was attributed to their lack of ability (Jungert et al., 2019; Sobieraj and Krämer, 2019). More recently, research in this area concerns the difference between males’ and females’ perceived abilities (Hand et al., 2017; Sobieraj and Krämer, 2019). Brown et al. (2017) reported instances where, although post-primary school students achieve the same grades in STEM subjects, females’ perceived ability was lower than that of males. Similarly, Sobieraj and Krämer investigated differences between male and female self-efficacy and perceived competence, finding that female students had lower self-perceptions of their abilities in STEM compared to male students. Hand et al. (2017) argued that the subtle biases of high school teachers have a detrimental effect on female self-efficacy in mathematics and science: such biases were evident when teachers expected girls and boys to perform differently in STEM subjects based on their perceptions of masculine and feminine traits. Ertl et al. (2017) reported that stereotypes have a damaging impact on female students’ self-concept, even when they have academic success. Ertl et al. suggested the reason may derive from the stereotypical belief that female achievements are due to diligence rather than ability. Kessels (2015) noted that when the stereotype is associated with a particular STEM career that does not align with a student’s self-concept, this constrains their career choice. Van Aalderen-Smeets and van der Molen (2018) hypothesised that changing students’ implicit theories of intelligence might improve their STEM-related self-efficacy beliefs and possibly the likelihood of choosing STEM-related study or careers. This is particularly important for female students, who are thought to hold entity beliefs more so than males; in other words, girls are more likely to believe that intelligence is fixed rather than malleable.

Overall, the literature identifies that females have lower levels of interest in STEM and lower perceptions of their abilities in STEM than males. This difference may be caused by social factors which influence students’ interest in STEM.

### Gender differences in STEM stereotypes

Blažev et al.’s (2017) study with school pupils in Croatia shows that male students and those who had previous success in STEM subjects are more likely to hold stereotypical beliefs about STEM. Several factors have been proposed to positively impact stereotypical beliefs, such as the presence of females in a class (Gunderson et al., 2012; Master et al., 2014; Riegle-Crumb et al., 2017). Riegle-Crumb et al. (2017) conducted a study regarding the presence of females in high school classes. They reported that female peers had a positive impact in reducing male peers’ stereotypical beliefs. The presence of female teachers seemed to have a similar impact: Master et al. (2014) found that female teachers reduced female students’ concerns about being negatively stereotyped in classroom situations. In contrast, Gunderson et al. (2012) reported that gender-biased stereotypes about females’ mathematics capabilities are cultivated, rather than ameliorated, by teachers. Exposure to role models is often promoted as a way of overcoming negative stereotypical beliefs about STEM. Gladstone and Cimpian’s (2021) systematic review of the literature in this field yielded four recommendations for maximising the effectiveness of role models in STEM for motivating students from diverse gender and ethnic backgrounds: (1) portray role models as being competent and successful, while avoiding extreme levels of success that might instead be alienating; (2) portray role models as being meaningfully similar to students; (3) prioritise exposure to role models from groups that are traditionally underrepresented in STEM; (4) portray role models’ success as being attainable. Luo et al. (2021) investigated upper primary students’ stereotypical beliefs about STEM careers and found that these beliefs negatively predicted STEM self-efficacy and career-related outcome expectations. Their findings suggest that interventions targeting STEM career aspirations need to target STEM stereotypes, self-efficacy, and outcome expectations simultaneously.

## Methodology

The data presented in this paper were collected as part of the STEMChAT project between 2019 and 2020 in Ireland. In line with the UNESCO (2017) Ecological Framework, while this paper focuses on specific psychological factors at the individual level as described, we are cognizant of the various other factors at other levels in which our study is contextualized. For example, at the family and peer level, our participants were sampled from schools located in socially disadvantaged areas, meaning that many of the students were likely from families with a lower socio-economic status which has been linked to lower academic achievement and expectations as well as possible adherence to more conventional gender role beliefs (Tenenbaum and Leaper, 2003; Organization for Economic Co-operation and Development [OECD], 2016). At the school level, participants attended 12 different post-primary schools with consequential exposure to different learning environments, including teacher quality and instructional practices, resources, assessments and school environments (UNESCO, 2017). At the societal level, deeply embedded societal and cultural norms regarding ‘traditional’ gendered subject choice at school and perceived gender ‘appropriate’ careers permeate more recent gender equality and inclusive policies in relation to STEM education and the STEM workforce (DES, 2017). As the project aimed to encourage female post-primary students to pursue STEM subjects and careers, the participating schools were selected according to their accessibility, social disadvantage of the area, and enrolment of female students.

### Participants

The participants of the study were post-primary students (mainly Transition Year students) in Ireland. Transition Year is a one-year program that students can volunteer to complete between Junior Cycle and Senior Cycle. In some Irish schools, Transition Year is mandatory. Each school designs its own Transition Year programme; therefore, programme content can vary between schools. A total of 308 students completed the survey including 218 females (71%) and 89 males (29%). One student did not disclose gender. Participants were aged between 14 and 18 years with a mean age of 16 years. Participants were sampled from schools participating in the STEMChAT project which led to a sampling bias in favour of females; this is discussed later as a potential limitation of the study.

### Study context and design

The surveys were completed by students from 12 post-primary schools (*n*_female school_ = 4, *n*_male school_ = 2, *n*_mixed school_ = 6) with 4 schools offering subjects from each of the STEM disciplines (science, technology, engineering and mathematics), 2 schools offering science, technology and mathematics subjects, and 6 schools offering only science and mathematics subjects. It is worth noting here that none of the single-sex girls’ schools were offering technology or engineering subjects.

In the Junior Cycle (lower secondary) curriculum, mathematics and science are taught as stand-alone subjects, and there is a suite of “technology” subjects comprising applied technology, engineering, wood technology, and graphics. There is a wider range of STEM subjects offered in the Senior Cycle curriculum, reflecting increased specialisation at this level of schooling. In their analysis of the treatment of STEM subjects within the Irish post-primary school context, McGarr and Lynch (2017) observed that mathematics and science occupy a higher status in the hierarchy of school subjects, in part because they have clearly defined subject boundaries and draw on long-established bodies of academic knowledge. In contrast, the traditionally vocational engineering and technology subjects hold a lower social status, draw on subject knowledge from a range of disciplines and are thus more loosely framed. This classification proved to be significant for our study, since the naming of the engineering and technology subjects made it difficult for students to identify them as belonging to the STEM categories of “engineering” and especially “technology.”

### Survey instrument

Our study is concerned with individual level factors referred to in the UNESCO (2017) Ecological Framework, specifically interest, perceived ability, value and commitment to STEM. These individual psychological factors are captured for both male and female students in our study, enabling gender comparison. We used a survey to identify students’ perceptions about STEM careers and students’ attitudes towards STEM. The items of the survey were drawn from two pre-existing validated surveys; Students’ Attitudes Towards STEM Survey (Mahoney, 2010) and STEM Semantics Survey (Tyler-Wood et al., 2010).

The Students’ Attitudes Towards STEM Survey (Mahoney, 2010) involved 24 items aiming to investigate high-school students’ awareness of (initial interest in) STEM, perceived ability in STEM, perceptions of the value of STEM, and commitment to (long-term interest in) STEM. For each item, students were asked to choose either science, technology, engineering or mathematics and indicate their response. For example, the first item, in the category of awareness of STEM, was “I do not like […]”; students responded by identifying one of the STEM disciplines that they did not like. Thus, for each item, students chose one STEM discipline that matched the attitude portrayed by that item: they were not required to indicate their attitudes towards every STEM discipline on every survey item.

The STEM semantics survey (Tyler-Wood et al., 2010) included semantic differential scales comprising five adjective pairs that reflect perceptions of science, technology, engineering and mathematics, respectively. A fifth scale, using the same adjective pairs, elicited perceptions of STEM career interests. Thus, the survey consisted of 25 items (five adjective pairs x five target areas). Students selected a response on a 1–7 scale to indicate how they felt about each content area. The wording of items was reviewed in terms of age-appropriateness and cultural differences. Only one word “mundane” was changed to “dull” to ensure students would understand the intended meaning.

### Data collection

Data collection took place in the Spring semester of 2018–2019 academic year (January–May) and in the Autumn semester of 2019–2020 academic year (September–December). Consent was obtained from the school principals, teachers who were providing their class time, students who volunteered to participate, and the guardians of participating students. The survey was completed at the beginning of a session for the STEMChAT project and students were asked to complete the survey to identify their pre-existing attitudes towards STEM.

### Data analysis

The survey data were analyzed through the Statistical Package for the Social Sciences (SPSS version 26). Data analysis comprised descriptive statistics, including response frequencies and percentages for both attitudinal items from Mahoney (2010) and STEM Semantics Survey items from Tyler-Wood et al. (2010), and median and interquartile range values for the STEM Semantic Survey items. The Kruskal–Wallis H test for differences between genders was also employed for the STEM Semantic Survey items but not for attitudinal items as the test can only be applied to continuous or ordinal variables. Because the attitudinal items used a nominal scale, it was neither possible nor meaningful to measure reliability *via* internal consistency. Reliability of the semantic differential scale was measured by calculating Cronbach’s alpha for each of the five component sub-scales (replicating the reliability analysis conducted by Tyler-Wood et al., 2010). Each sub-scale had a high level of internal consistency as determined by the Cronbach’s alpha results shown in Table 1. The internal consistency of the scale could not be improved by removing any of the items.

**Table 1:** Internal consistency reliabilities for STEM semantics scales.

Scale	Number of items	Alpha
Science	5	0.841
Mathematics	5	0.833
Engineering	5	0.886
Technology	5	0.887
STEM career	5	0.810

## Findings

### Context of the study

Participants were asked whether their school offered each of the STEM subjects and which STEM subjects they studied. Responses indicated that all schools offered science and mathematics subjects and only 4 schools offered engineering, but there was some confusion amongst participants as to whether technology was offered as a subject in their school (subjects actually offered by each school were confirmed by the authors and are provided in the Methodology section). This may be indicative of students’ confusion about what technology means: for example, they may have interpreted technology as meaning ICT or computer science as opposed to construction studies or design and communication graphics, even though ICT/computer science was not a subject in the school curriculum at the time of this study. Frequency of responses for males and females studying each of the STEM disciplines are shown in Table 2. Percentages by gender were calculated based on total females or males. For example, 32 females correspond to 14.68% of female participants.

**Table 2:** STEM subjects studied by gender.

	Science, *N* (%)	Technology, *N* (%)	Engineering, *N* (%)	Mathematics, *N* (%)
Females	206 (94.5%)	32 (14.7%)	25 (11.5%)	218 (100.0%)
Males	79 (88.8%)	26 (29.2%)	18 (20.2%)	87 (97.8%)
Total	286 (92.9%)	58 (18.8%)	43 (14.0%)	306 (99.4%)

Apart from two students, all participants studied mathematics and the majority studied science, with a slightly greater percentage of females compared to males studying science. Males were almost twice as likely as females to be studying technology and engineering, but the number of participants studying these subjects was considerably lower than mathematics and science. It should be recalled that mathematics is effectively compulsory in Irish post-primary schools and science is compulsory in the majority of Irish post-primary schools at Junior Cycle. When considering students’ attitudes and perceptions as reported in the following section, we need to bear in mind the relatively low numbers of students who have experienced technology and engineering which will feasibly have impacted their responses to the survey items. In particular, when participants were asked to choose one of science, technology, engineering or mathematics when responding to Mahoney’s (2010) Attitudes toward STEM items, some participants may have been chosen only from those subjects they have personally experienced in school or may have indifferent attitudes towards subjects (such as technology/engineering subjects) in which they have had little or no experience. Nevertheless, the different response patterns, indicating that students may be less familiar with or interested in technology and engineering subjects, constitute a significant finding from the study and raise questions about the relative status and visibility of the constituent STEM disciplines in Irish post-primary schools.

### Students’ attitudes towards STEM

Participants’ attitudes to STEM were measured using Mahoney’s (2010) instrument, where participants select either science, technology, engineering or mathematics for each of the items. Frequency of responses for each item across the four options was obtained for all participants and for females and males. Frequencies for the items intended to capture participants’ initial interest in STEM are shown in Table 3. Negatively worded items are labelled (N) and positively worded items are labelled (P). The response option with the highest frequency is shaded. For each item there were some participants who did not respond or who gave multiple responses and these participants are shown in the last column of the table.

**Table 3:** Initial interest in STEM.

Item	Science, *N* (%)	Technology, *N* (%)	Engineering, *N* (%)	Mathematics, *N* (%)	Not given/multiple responses, *N* (%)
*I do not like […] (N)*					
Female	31 (14.2%)	34 (15.6%)	57 (26.1%)	89 (40.8%)	7 (3.2%)
Male	26 (29.2%)	8 (9.0%)	13 (14.6%)	33 (37.1%)	9 (2.0%)
All	58 (18.8%)	42 (13.6%)	70 (22.7%)	122 (39.6%)	16 (5.2%)
*I enjoy learning about […] (P)*					1 ((0.5%)
Female	147 (67.4%)	27 (12.4%)	12 (5.5%)	31 (14.2%)	
Male	32 (36.0%)	20 (22.5%)	18 (20.2%)	16 (18.0%)	3 (3.4%)
All	179 (58.1%)	47 (15.3%)	30 (9.7%)	47 (15.3%)	5 (1.6%)
*I am curious about […] (P)*					
Female	80 (36.7%)	66 (30.3%)	59 (27.1%)	11 (5.0%)	2 (0.9%)
Male	23 (25.8%)	31 (34.8%)	24 (27.0%)	7 (7.9%)	4 (4.5%)
All	103 (33.4%)	97 (31.5%)	83 (26.9%)	18 (5.8%)	7 (2.2%)
*I am not interested in […] (N)*					
Female	19 (8.7%)	62 (28.4%)	78 (35.8%)	47 (21.6%)	12 (5.5%)
Male	29 (32.6%)	13 (14.6%)	15 (16.9%)	27 (30.3%)	5 (5.6%)
All	48 (15.6%)	75 (24.4%)	93 (30.2%)	74 (24.0%)	18 (5.9%)
*I like […] (P)*					
Female	89 (40.8%)	36 (16.5%)	14 (6.4%)	71 (32.6%)	8 (3.7%)
Male	23 (25.8%)	18 (20.2%)	22 (24.7%)	24 (27.0%)	2 (2.2%)
All	112 (36.4%)	54 (17.5%)	36 (11.7%)	95 (30.8%)	11 (3.6%)
*[…] is appealing to me (P)*					
Female	96 (44.0%)	47 (21.6%)	34 (15.6%)	35 (16.1%)	6 (2.8%)
Male	25 (28.1%)	14 (15.7%)	34 (38.2%)	12 (13.5%)	4 (4.5%)
All	121 (39.3%)	61 (19.8%)	68 (22.1%)	47 (15.3%)	11 (3.5%)

Participants’ responses regarding initial interest in STEM show the most interest in science. In particular, majority of students stated that they enjoyed learning about science, although it is clear from the gender breakdown that more females are interested in science compared to males. Mathematics was the subject least liked by both male and female participants with almost 40% of participants stating that they do not like mathematics. There might be different reasons for this finding, such as students’ mathematics self-efficacy, stereotypical beliefs about mathematics being for intelligent people, and students perceiving mathematics as numbers rather than understanding its role in real life. Bearing in mind that only 18.8% of participants were studying technology subjects and 14.0% engineering, it was noteworthy to see the high proportion (31.5%) who identified technology as the STEM discipline they were curious about and engineering as that having the least interest for them (30.2%).

Frequencies for the items measuring participants’ perceived ability in STEM are shown in Table 4. Negatively worded items are labelled (N) and positively worded items are labelled (P). The response option with the highest frequency is shaded for each item.

**Table 4:** Perceived ability in STEM.

Item	Science, *N* (%)	Technology, *N* (%)	Engineering, *N* (%)	Mathematics, *N* (%)	Not given/multiple responses, *N* (%)
*[…] is difficult for me (N)*					
Female	35 (16.1%)	22 (10.1%)	26 (11.9%)	129 (59.2%)	6 (2.7%)
Male	26 (29.2%)	6 (6.7%)	5 (5.6%)	48 (53.9%)	4 (4.5%)
All	61 (19.8%)	28 (9.1%)	31 (10.1%)	177 (57.5%)	11 (3.5%)
*I do well in […] (P)*					
Female	115 (52.8%)	10 (4.6%)	8 (3.7%)	75 (34.4%)	10 (4.6%)
Male	25 (28.1%)	10 (11.2%)	13 (14.6%)	36 (40.4%)	5 (5.6%)
All	140 (45.5%)	20 (6.5%)	21 (6.8%)	111 (36.0%)	16 (5.1%)
*I am not confident about my work in […] (N)*					
Female	50 (22.9%)	30 (13.8%)	27 (12.4%)	103 (47.2%)	8 (3.7%)
Male	29 (32.6%)	12 (13.5%)	6 (6.7%)	34 (38.2%)	8 (9.0%)
All	79 (25.6%)	42 (13.6%)	33 (10.7%)	137 (44.5%)	17 (5.5%)
*I have a hard time in […] (N)*					
Female	43 (19.7%)	28 (12.8%)	29 (13.3%)	109 (50.0%)	9 (4.1%)
Male	30 (33.7%)	3 (3.4%)	5 (5.6%)	44 (49.4%)	7 (7.9%)
All	74 (24.0%)	31 (10.1%)	34 (11.0%)	153 (49.7%)	16 (5.2%)
*Assigned work in […] is easy for me (P)*					
Female	118 (54.1%)	19 (8.7%)	10 (4.6%)	63 (28.9%)	8 (3.7%)
Male	26 (29.2%)	13 (14.6%)	12 (13.5%)	36 (40.4%)	2 (2.2%)
All	144 (46.8%)	32 (10.4%)	22 (7.1%)	100 (32.5%)	10 (3.2%)
*I cannot figure out […] (N)*					
Female	33 (15.1%)	46 (21.1%)	48 (22.0%)	84 (38.5%)	7 (3.2%)
Male	23 (25.8%)	15 (16.9%)	11 (12.4%)	29 (32.6%)	11 (12.4%)
All	57 (18.5%)	61 (19.8%)	59 (19.2%)	113 (36.7%)	18 (5.8%)

Supporting the finding about students’ attitude, the results showed that students’ perceived ability in mathematics was low. A clear majority of participants stated that mathematics was difficult for them, that they were not confident about their work in mathematics, that they have a hard time in and cannot figure out mathematics. Similar to the initial interest items, science was the most frequent response option for positively worded items for females, although mathematics was the subject in which male participants most frequently reported that they do well. Unsurprisingly, science and mathematics were the two most frequently chosen response options for most of the items in relation to participants’ perceived ability, likely due to the fact that most students do not study technology or engineering. The data also showed that while the female participants’ perceived ability in technology and engineering was very low, a much higher percentage of male participants said they did well in these subjects and found the work easy.

Frequencies for the items measuring participants’ perceptions of the value of STEM are shown in Table 5. Negatively worded items are labelled (N) and positively worded items are labelled (P). The response option with the highest frequency is shaded for each item.

**Table 5:** Value of STEM.

Item	Science, *N* (%)	Technology, *N* (%)	Engineering, *N* (%)	Mathematics, *N* (%)	Not given/multiple responses, *N* (%)
*[…] is important to me (P)*					
Female	95 (43.6%)	24 (11.0%)	9 (4.1%)	75 (34.4%)	15 (6.9%)
Male	26 (29.2%)	13 (14.6%)	15 (16.9%)	31 (34.8%)	4 (4.5%)
All	121 (39.3%)	37 (12.0%)	24 (7.8%)	107 (34.7%)	19 (6.1%)
*I feel there is a need for […] (P)*					
Female	79 (36.2%)	38 (17.4%)	20 (9.2%)	73 (33.5%)	8 (3.7%)
Male	25 (28.1%)	12 (13.5%)	17 (19.1%)	31 (34.8%)	4 (4.5%)
All	104 (33.8%)	50 (16.2%)	37 (12.0%)	105 (34.1%)	12 (3.9%)
*I do not need […] (N)*					
Female	24 (11.0%)	67 (30.7%)	89 (40.8%)	21 (9.6%)	17 (7.8%)
Male	25 (28.1%)	20 (22.5%)	29 (32.6%)	6 (6.7%)	9 (10.1%)
All	49 (15.9%)	87 (28.2%)	118 (38.3%)	27 (7.8%)	27 (8.8%)
*It is valuable for me to learn […] (P)*					
Female	93 (42.7%)	13 (6.0%)	8 (3.7%)	96 (44.0%)	8 (3.6%)
Male	20 (22.5%)	12 (13.5%)	8 (9.0%)	46 (51.7%)	3 (3.3%)
All	114 (37.0%)	25 (8.1%)	16 (5.2%)	142 (46.1%)	11 (3.5%)
*[…] is good for me (P)*					
Female	95 (43.6%)	17 (7.8%)	7 (3.2%)	88 (40.4%)	11 (5.1%)
Male	37 (41.6%)	9 (10.1%)	12 (13.5%)	28 (31.5%)	3 (3.4%)
All	133 (43.2%)	28 (8.4%)	19 (6.2%)	116 (37.7%)	14 (4.5%)
*I do not care about […] (N)*					
Female	19 (8.7%)	67 (30.7%)	79 (36.2%)	41 (18.8%)	12 (5.5%)
Male	26 (29.2%)	18 (20.2%)	27 (30.3%)	9 (10.1%)	9 (10.1%)
All	45 (14.6%)	85 (27.6%)	106 (34.4%)	50 (16.2%)	22 (7.2%)

Responses to the attitudinal items regarding value of STEM demonstrate high value of both science and mathematics as important and valuable to learn, although again male participants responded more positively towards mathematics than female participants. The responses indicate that although students may have lower interest in mathematics and have poor perceptions of their mathematical ability, they recognize the value of learning mathematics. There was a strong perception amongst participants that engineering, and to a lesser extent technology, were subjects they did not need and did not care about. As many participants had not experienced either of these subject areas, their responses highlight the difficulty of promoting positive attitudes to the full range of STEM subjects when there is not universal and equitable access to these subjects in all schools.

The final group of attitudinal items from Mahoney (2010) measuring commitment or long-term interest in STEM are shown in Table 6. Negatively worded items are labelled (N) and positively worded items are labelled (P). The response option with the highest frequency is shaded for each item.

**Table 6:** Long-term interest in STEM.

Item	Science, *N* (%)	Technology, *N* (%)	Engineering, *N* (%)	Mathematics, *N* (%)	Not given/multiple responses, *N* (%)
*I will continue to enjoy […] (P)*					
Female	128 (58.7%)	27 (12.4%)	12 (5.5%)	45 (20.6%)	6 (2.8%)
Male	24 (27.0%)	18 (20.2%)	23 (25.8%)	19 (21.3%)	5 (5.6%)
All	152 (49.4%)	45 (14.6%)	35 (11.4%)	64 (20.8%)	12 (3.8%)
*I am not interested in a career involving […] (N)*					
Female	25 (11.5%)	37 (17.0%)	91 (41.7%)	47 (21.6%)	18 (8.2%)
Male	36 (40.4%)	13 (14.6%)	22 (24.7%)	11 (12.4%)	7 (7.8%)
All	61 (19.8%)	50 (16.2%)	113 (36.7%)	58 (18.8%)	26 (8.4%)
*I am interested in alternative programs in […] (P)*					
Female	110 (50.5%)	37 (17.0%)	23 (10.6%)	33 (15.1%)	15 (6.9%)
Male	32 (36.0%)	20 (22.5%)	18 (20.2%)	13 (14.6%)	6 (6.7%)
All	143 (46.4%)	57 (18.5%)	41 (13.3%)	46 (14.9%)	21 (6.8%)
*I would like to learn more about […] (P)*					
Female	94 (43.1%)	60 (27.5%)	42 (19.3%)	13 (6.0%)	9 (4.1%)
Male	25 (28.1%)	27 (30.3%)	23 (25.8%)	12 (13.5%)	2 (2.2%)
All	119 (38.6%)	87 (28.2%)	65 (21.1%)	26 (8.4%)	11 (3.5%)
*I do not wish to continue my education in […] (N)*					
Female	36 (16.5%)	49 (22.5%)	47 (21.6%)	59 (27.1%)	27 (12.4%)
Male	31 (34.8%)	14 (15.7%)	15 (16.9%)	16 (18.0%)	13 (14.6%)
All	67 (21.8%)	63 (20.5%)	62 (20.1%)	75 (24.4%)	41 (13.3%)
*I am committed to learning […] (P)*					
Female	100 (45.9%)	9 (4.1%)	9 (4.1%)	83 (38.1%)	17 (7.8%)
Male	22 (24.7%)	13 (14.6%)	15 (16.9%)	34 (38.2%)	5 (5.6%)
All	122 (39.6%)	22 (7.1%)	24 (7.8%)	118 (38.3%)	22 (7.1%)

Responses to the items regarding long-term interest in STEM show similarity with initial interest as females’ responses were overwhelmingly positive towards science with more than half indicating that they would continue to enjoy science and are interested in alternative programs. However, there were distinctive gender differences in students’ interest in science. Males were least interested in a career involving science, were more committed to learning mathematics, and curious to learn more about technology. Technology was the second most frequently selected option in relation to interest in alternative programs for both males and females. Engineering was overwhelmingly the field in which participants, especially females, were not interested in pursuing a career. As for the previous set of items on the value of STEM, lack of familiarity with and exposure to engineering subjects at school may have influenced the responses.

### Perceptions of STEM disciplines and careers

The second part of our survey included Tyler-Wood et al.’s (2010) STEM Semantics Scale where participants select a rating between 1 and 7 for five adjective pairs. Three of the adjective pairs are ordered positive–negative and two adjective pairs are ordered negative–positive. Therefore, for the positive–negative pairs, a lower score indicates a positive response while a higher score indicates a negative response. For the negative–positive pairs, a lower score indicates a negative response while a higher score indicates a positive response. Median scores and Interquartile range values for each adjective pair was calculated for each of the STEM disciplines as well as STEM careers as shown in Table 7.

**Table 7:** Median and interquartile range for STEM semantics survey.

Adjective pair	Science (IQR Q1:Q3)	Mathematics (IQR Q1:Q3)	Engineering (IQR Q1:Q3)	Technology (IQR Q1:Q3)	STEM career (IQR Q1:Q3)
*Fascinating – Dull*	2.0 (1.0:4.0)	4.0 (3.0:6.0)	4.0 (2.0:6.0)	4.0 (2.0:5.0)	3.0 (2.0:4.0)
*Appealing – Unappealing*	3.0 (2.0:4.0)	4.0 (3.0:6.0)	4.0 (2.0:6.0)	3.0 (2.0:5.0)	3.0 (1.0:4.0)
*Exciting – Unexciting*	3.0 (2.0:4.0)	4.0 (3.0:6.0)	4.0 (3.0:6.0)	4.0 (3.0:5.0)	3.0 (2.0:4.0)
*Means Nothing – Means a Lot*	6.0 (4.0:7.0)	5.0 (4.0:6.75)	4.0 (3.0:5.0)	5.0 (3.0:6.0)	5.0 (4.0:7.0)
*Boring – Interesting*	5.0 (4.0:7.0)	4.0 (2.0:6.0)	4.0 (3.0:6.0)	4.0 (3.0:6.0)	5.0 (4.0:7.0)

With regards to perceptions of science, the median scores for the five items indicate that participants generally responded positively with science being perceived as fascinating, somewhat appealing, somewhat exciting, meaning a lot and somewhat interesting. Looking at students’ attitude, perceived ability, value and long-term interest, this result may not be surprising. Median scores for the five items in relation to mathematics, engineering and technology were generally more neutral compared to perceptions of science so the interquartile range is used to determine whether responses tend towards the positive or negative perception. Participants’ perceptions rated mathematics as neutral but tending towards dull, unappealing, unexciting, somewhat meaningful, and interesting. Students perceived engineering neutral for three adjective pairs but tending towards unexciting and interesting. Participants perceived technology as neutral but tending towards dull, somewhat appealing, somewhat meaningful and interesting. Median scores for the five items regarding perceptions of STEM careers were slightly positive with a STEM career perceived as somewhat fascinating, somewhat appealing, somewhat exciting, somewhat meaningful and somewhat interesting.

To compare differences between genders for the STEM Semantics Scale, we used the Kruskal–Wallis H-Test since there was a considerable difference between the number of participants in the comparative groups (*n*_female_ = 218; *n*_male_ = 89). As the distributions of females’ and males’ scores were not identical, we cannot compare medians and instead report on mean ranks. Results for differences between genders are shown in Appendix 1.

With regards to participants’ perceptions of science, there was a statistically significant difference (p<0.05) between genders for all five items as follows:

- In rating science as fascinating – dull, $\chi^{2}\left(1\right)$ = 6.002, p = 0.014 with a mean rank score of 143.02 for females and 169.42 for males.
- In rating science as appealing – unappealing, $\chi^{2}\left(1\right)$ = 14.044, p = 0.000 with a mean rank score of 137.11 for females and 177.23 for males.
- In rating science as exciting – unexciting, $\chi^{2}\left(1\right)$ = 5.901, p = 0.015 with a mean rank score of 141.41 for females and 167.63 for males.
- In rating science as means nothing – means a lot, $\chi^{2}\left(1\right)$ = 5.729, p = 0.017 with a mean rank score of 155.93 for females and 130.35 for males.
- In rating science as boring – interesting, $\chi^{2}\left(1\right)$ = 6.243, p = 0.012 with a mean rank score of 155.73 for females and 128.91 for males.

These results indicate that females in this study had significantly more positive perceptions of science compared to males for all items, with the greatest difference between the genders found in their rating of science in terms of appealing – unappealing.

Comparing females’ and males’ perceptions of mathematics, there was a statistically significant difference (p<0.05) between genders in rating mathematics as boring – interesting, $\chi^{2}$(1) = 4.638, p = 0.031 with a mean rank score of 143.25 for females and 166.72 for males. There was no statistically significant difference (p<0.05) between genders in rating other mathematics items. These results indicate that males have a significantly greater interest in mathematics compared to females in our study, but while male participants had more positive responses to mathematics compared to females for other items the differences between the genders’ responses were not statistically significant.

In relation to participants’ perceptions of engineering, a statistically significant difference (p<0.05) between genders was found for four of the scale items as follows:

- In rating engineering as fascinating – dull, $\chi^{2}\left(1\right)$ = 12.133, p = 0.000 with a mean rank score of 158.97 for females and 121.34 for males.
- In rating engineering as appealing – unappealing, $\chi^{2}\left(1\right)$ = 15.977, p = 0.000 with a mean rank score of 160.84 for females and 117.35 for males.
- In rating engineering as exciting – unexciting, $\chi^{2}\left(1\right)$ = 11.003, p = 0.001 with a mean rank score of 157.45 for females and 121.85 for males.
- In rating engineering as boring – interesting, $\chi^{2}\left(1\right)$ = 5.255, p = 0.022 with a mean rank score of 139.90 for females and 164.66 for males.

These results indicate that males in our study had significantly more positive perceptions of engineering compared to females for four items, with the greatest difference between the genders was found in their rating of engineering in terms of appealing – unappealing.

There was a statistically significant difference (p<0.05) between genders found for three of the scale items in relation to technology as follows:

- In rating technology as fascinating – dull, $\chi^{2}\left(1\right)$ = 6.485, p = 0.011 with a mean rank score of 157.05 for females and 129.56 for males.
- In rating technology as appealing – unappealing, $\chi^{2}\left(1\right)$ = 7.718, p = 0.005 with a mean rank score of 157.08 for females and 126.83 for males.
- In rating technology as exciting – unexciting, $\chi^{2}\left(1\right)$ = 9.670, p = 0.002 with a mean rank score of 157.68 for females and 124.08 for males.

These results indicate that male participants in our study had significantly more positive responses to technology compared to females for these three items, with the greatest difference between the genders found in their rating of technology in terms of being exciting – unexciting. No statistically significant difference emerged between the genders’ responses to the items means nothing – means a lot or boring – interesting.

There was no statistical difference (p<0.05) between genders in rating any of the career items.

## Discussion

In this section, we discuss the findings to address our research questions: 1. What are student attitudes towards science, technology, engineering and mathematics as measured through: awareness (initial interest); perceived ability; value; and commitment (long-term interest)? 2. What gender differences occur regarding students’ attitudes to science, technology, engineering and mathematics? In addressing these questions, our discussion is centred around three key issues and/or challenges: access to STEM, attitudes to STEM and gender differences. We also discuss limitations of our study.

### Access to STEM subjects

Our findings highlight the differential access to STEM subjects for post-primary students in Ireland, particularly in relation to technology and engineering subjects. Students attending single-sex schools in this study did not have access to engineering subjects at their school and many students were confused as to whether they had access to technology subjects due to the lack of clarity in labelling of these subjects in the school curriculum. Post-primary schools in Ireland fall into three categories: voluntary secondary schools, vocational schools, and community and comprehensive schools (Eurydice, 2019). Single-sex schools are secondary schools while co-educational schools include all three school types. Traditionally, secondary schools chiefly offered perceived ‘academic’ subjects while vocational schools offered more ‘practical’ subject choices and this traditional segregation continues to some extent today. As a result, students attending secondary schools, and consequentially students attending single-sex schools, have limited access to subjects from the technology and engineering suite. Interpreting this situation in terms of the UNESCO (2017) Ecological Framework, we see how school and societal factors impact individual factors in terms of females’ participation, achievement and progression in STEM studies and careers. Many students do not have a choice in relation to the post-primary school they attend, meaning that access to all STEM subjects is essentially a ‘postcode lottery’. Students, or indeed their parents, who have a choice and prefer single-sex educational settings may or may not be aware of the limiting consequences of their choice. If the gender gap problem is considered as a “leaky pipeline,” with low female participation in second-level STEM subjects leading to similarly low participation rates in third level STEM programs, lack of access to STEM subjects must be seen as one of the sources of the leaks and a key challenge to students, and for the purpose of our interest particularly, females’ participation in STEM education and careers. “Education systems and schools play a central role in determining girls’ interest in STEM subjects and in providing equal opportunities to access and benefit from quality STEM education” (UNESCO, 2017, p. 11), but clearly there is significant scope for improving equal opportunities in the current Irish post-primary system.

Furthermore, there is a need for greater clarity about what STEM means in relation to post-primary education. Our study found considerable confusion among students about what STEM means in relation to school subjects, particularly technology. This could be observed in the fact some students were unsure whether technology was offered at their school. At Junior Cycle in Ireland (which all participants in our study had completed), there are two technology subjects – construction studies and design and communication graphics. However, even at a state curricular level, there appears to be a lack of clarity about what technology constitutes. The National Council for Curriculum and Assessment (NCCA) includes engineering in the technology suite of subjects, which highlights a systemic lack of clarity concerning the technology and engineering aspects of STEM in the Irish school system. McGarr and Lynch (2017) discussed the absence of technology and engineering from the broader STEM agenda in post-primary schools, pointing not only to the under-resourced and out of date subjects on offer but also to the social history of these vocationally-oriented subjects and their resultant lower status in the school curriculum. Our findings indicate that this ‘absence’ manifests in lack of understanding and awareness of these subjects among students. This leads us to suggest that, in Ireland, the STEM education agenda might be better represented as S(t)eM because of the limited offering of engineering subjects and the confusion that exists about the meaning of “technology.” Adopting engineering design as a catalyst to STEM education and developing a more profound and clear understanding of technology is therefore paramount to STEM education (Kelley and Knowles, 2016). With different interpretations of STEM amongst researchers (Bybee, 2013) and between contexts (research, policy, school, society, media etc.), we argue for the need for greater transparency and consistency about what STEM means, especially in relation to school subjects.

### Student attitudes to STEM

Students in our study generally held positive attitudes towards science (enjoyment, curiosity, liking, importance, value, doing well, commitment to learning) and less positive attitudes towards mathematics (not liking, difficult, not confident, do not understand) while still endorsing the value of mathematics and a commitment to learning it. Students in our study also expressed a lack of interest in engineering, and no strong views on technology, possibly because they did not understand what this discipline meant in a school context which presents a challenge to future participation. Engineering was perceived as having the least value among the STEM subjects, which again could be linked to the lack of access in schools compared to science and mathematics and the sub-cultures surrounding STEM subjects in post-primary education in Ireland (McGarr and Lynch, 2017). This finding highlights again the interrelation of school and societal factors with the individual’s STEM ecosystem (UNESCO, 2017). Ireland’s *STEM Education Policy Statement 2017–2026* advocates for “the highest quality STEM education experience for learners that nurtures curiosity, inquiry, problem-solving, creativity, ethical behaviour, confidence and persistence, along with the excitement of collaborative innovation” (DES, 2017, p. 12). The report highlights the importance of inspiring young people’s curiosity and the related educational benefits. Our study indicates that students are distinctly lacking this curiosity about mathematics, more so than any other STEM subject. Our study also found demonstrably lower confidence in mathematics compared to other STEM subjects, although this is caveated by the low participation in technology and engineering. We argue the need to identify and implement ways to foster students’ curiosity and confidence regarding mathematics as mathematics underpins all STEM learning. Additionally, there is an obvious need to introduce engineering and technology subjects to students to be able to determine their real attitude towards these subjects. Recent research has highlighted the importance of value-related beliefs as a strong predictor of career aspirations in STEM (Wang et al., 2013; Wegemer and Eccles, 2019). In our study, technology and engineering were perceived by students as having less value than science and mathematics, and this ‘lower value’ is also reflected in the challenges of reduced access to these subjects and the lack of clarity surrounding these subjects in the Irish post-primary system.

### Gender differences

In our study, females had significantly more positive perceptions and attitudes to science compared to males. Science was perceived by female students as the most valuable STEM subject to learn, more appealing, interesting and enjoyable and with higher levels of confidence in science compared to other STEM subjects. Our survey did not differentiate between biology, chemistry, physics, and agricultural science as individual science subjects, which may have elicited different responses. In light of the significant gender differences in science subject enrolments at Senior Cycle in Ireland, the question remains as to how to harness this interest in science reported by females in our study and apply it to the individual scientific disciplines such as physics and chemistry, as well as to other STEM subjects. What exactly is it about science at Junior Cycle that attracts female students and yet does not translate into studying the physical sciences at Senior Cycle and beyond? There is future work to be done in answering these questions.

Female students in our study reported less positive attitudes to mathematics compared to male students in terms of liking, interest and value. Mathematics was perceived by all students as the most difficult STEM subject, although female students reported less positive perceptions about their abilities compared to males. This finding ties in with the findings of Brown et al. (2017), who reported that in instances where post-primary school students achieve the same grades, females’ perceived ability in STEM subjects is lower than males. This lower perception of ability by females can be particularly potent in relation to future ability and may be a factor in females’ avoidance of future studies or careers in these disciplines. Female students in our study also reported lower enjoyment of engineering compared to male students, while females also had significantly less positive perceptions of technology compared to males. This is likely linked to these female students having little exposure to technology and engineering at school, although that challenge also exists for male students.

A positive finding in our study was that no significant difference emerged between male and female students’ perceptions of STEM careers. One possible explanation is that the participants were not yet at Senior Cycle and therefore had not decided on a career/college path. This may indicate the potential for interceding at this stage of post-primary education, prior to subject choice at Senior Cycle, with a view to enhancing awareness of STEM courses and careers and encouraging future participation and engagement with STEM. The Accenture (2014) report highlights that parents may have low career knowledge, which makes it difficult for them to offer career advice to their children, who in turn have trouble making decisions and understanding careers. Future work is required to design, implement and evaluate interventions aimed at students, particularly females, and potentially their parents to encourage informed decisions about future STEM subject/career choice.

### Limitations of the study

There are some limitations of our study which we recognise and outline here. Firstly, a sampling bias in favour of females occurred as only schools that participated in the STEMChAT project were sampled and the project purposefully targeted female students in particular. Therefore, some of the schools that participated were all-girls schools. However, there were sufficient numbers of male students to allow for statistically valid gender comparison. The inclusion of single-sex girls’ schools also highlighted the inequitable access to technology and engineering subjects experienced by female students. Secondly, the schools sampled in this study were all located in one province of Ireland. We do not claim that findings are representative of all students in Ireland. Rather, our findings provide some insight into these particular students’ attitudes towards STEM while bringing to light some challenges that exist regarding subject access, attitudes and gender differences which need further research on a national scale. Thirdly, although STEM was defined as science, technology, engineering and mathematics, what was meant by each subject was not interrogated in the survey. For example, in relation to the term science, some students may have been answering the survey based on a particular aspect of science (e.g., physics) or interpreting the term as encompassing many different aspects of science. There was evident confusion among some students about what technology means, as previously discussed. Future data collection should seek to clarify and unpack each of the STEM disciplines.

## Conclusion and recommendations

This paper aimed to identify students’ attitudes to STEM as well as any gender differences with regards to these attitudes, and ultimately to determine challenges to female students’ participation in STEM both at the post-primary (secondary) level and beyond. We surveyed 308 post-primary students in Ireland as part of a one-year SFI-funded research project “STEMChAT: Women as catalysts for change in STEM education.” The results pointed to three key findings and challenges relating to (1) access to STEM subjects, (2) students’ attitudes, and (3) gender differences, and we offer some recommendations in relation to these.

The results in this study highlight the challenge of equal access to STEM subjects in different types of post-primary schools. For example, many secondary schools, particularly the single-sex ones, have limited access to technology and engineering subjects. If our aim is to enhance the STEM subject and career involvement, the current Irish post-primary school system should be reformed to provide equal opportunities to all students, particularly girls, in order to access STEM subjects. Additionally, considerable confusion emerged among students about what STEM means regarding school subjects, particularly technology and engineering, which might be related to the access (or lack thereof) to these subjects in schools. STEM education stays as a contested term lacking a unified definition (Bybee, 2013; McLoughlin et al., 2020); we suggest developing greater transparency and consistency about what STEM means, especially in relation to school subjects.

Students in our study generally held positive attitudes towards science and less positive attitudes towards mathematics, while they expressed a lack of interest in engineering and no strong views on technology. Six schools (including 4 girls’ schools) did not offer access to technology and engineering subjects, which conceivably might have affected these results. We suggest identifying and implementing ways to foster students’ curiosity and confidence regarding mathematics and providing increased exposure to engineering and technology subjects to enable students to determine more informed attitudes towards these subjects.

Our study found that female students had significantly more positive attitudes towards science compared to males while, in comparison, males had a significantly more positive response to mathematics compared to females. Further research is needed to determine how to harness female students’ positive attitudes towards science and cultivate this in the other STEM subjects. Additionally, no significant difference was found in this study between male and female students’ perceptions of STEM careers. We suggest that an opportunity exists during Transition Year, prior to Senior Cycle subject choice, to enhance students’ awareness of STEM subjects, third-level courses and careers in order to encourage future participation and engagement with STEM.

Overall, this study contributes to the STEM education field by identifying challenges to female students’ participation in STEM in the Irish context and recommending future research to further understand and overcome them. In so doing, our paper contributes to the international discussion and agenda to provide “equal opportunities to access and benefit from quality STEM education” (UNESCO, 2017, p. 11).

## Data availability statement

The datasets presented in this article are not readily available because of participant confidentiality. Requests to access the datasets should be directed to CL: shaciaral@gmail.com.

## Ethics statement

The studies involving human participants were reviewed and approved by The University of Limerick Research Ethics Committee. Written informed consent to participate in this study was provided by the participants’ legal guardian/next of kin.

## Author contributions

CL contributed to data collection, led data analysis and interpretation, manuscript design, and writing and revision. SK-C and RK contributed to survey design, data collection, and manuscript writing and revision. TO’C contributed to data collection and manuscript writing and revision. MG contributed to survey design and manuscript revision. All authors read the final manuscript. All authors contributed to the article and approved the submitted version.

## Funding

The STEMChAT project was funded by a Science Foundation Ireland Discover Program Grant (Proposal ID 18/DP/5926). The funding body was not involved in the design, data collection, analysis, interpretation or writing of this manuscript.

## Conflict of interest

The authors declare that the research was conducted in the absence of any commercial or financial relationships that could be construed as a potential conflict of interest.

## Publisher’s note

All claims expressed in this article are solely those of the authors and do not necessarily represent those of their affiliated organizations, or those of the publisher, the editors and the reviewers. Any product that may be evaluated in this article, or claim that may be made by its manufacturer, is not guaranteed or endorsed by the publisher.

## Appendix

**Appendix 1. Kruskal–Wallis H-Test for differences between genders for STEM semantics scale.**

**Table:**

Item	Science	Mathematics	Engineering	Technology	STEM career
*Fascinating – Dull*	$\chi^{2}\left(1\right)$ = 6.002, p = 0.014	$\chi^{2}\left(1\right)$ = 2.814, p = 0.093	$\chi^{2}\left(1\right)$ = 12.133, p = 0.000	$\chi^{2}\left(1\right)$ = 6.485, p = 0.011	$\chi^{2}\left(1\right)$ = 0.389, p = 0.533
*Appealing – Unappealing*	$\chi^{2}\left(1\right)$ = 14.044, p = 0.000	$\chi^{2}\left(1\right)$ = 1.709, p = 0.191	$\chi^{2}\left(1\right)$ = 15.977, p = 0.000	$\chi^{2}\left(1\right)$ = 7.718, p = 0.005	$\chi^{2}\left(1\right)$ = 0.301, p = 0.584
*Exciting – Unexciting*	$\chi^{2}\left(1\right)$5.901, p = 0.015	$\chi^{2}\left(1\right)$ = 1.380, p = 0.240	$\chi^{2}\left(1\right)$ = 11.003, p = 0.001	$\chi^{2}\left(1\right)$ = 9.670, p = 0.002	$\chi^{2}\left(1\right)$ = 0.008, p = 0.927
*Means Nothing – Means a Lot*	$\chi^{2}\left(1\right)$ = 5.729, p = 0.017	$\chi^{2}\left(1\right)$ = 1.263, p = 0.261	$\chi^{2}\left(1\right)$ = 3.049, p = 0.081	$\chi^{2}\left(1\right)$ = 0.841, p = 0.359	$\chi^{2}\left(1\right)$ = 0.134, p = 0.715
*Boring – Interesting*	$\chi^{2}\left(1\right)$ = 6.243, p = 0.012	$\chi^{2}\left(1\right)$ = 4.638, p = 0.031	$\chi^{2}\left(1\right)$ = 5.255, p = 0.022	$\chi^{2}\left(1\right)$ = 2.288, p = 0.130	$\chi^{2}\left(1\right)$ = 0.003, p = 0.958

## Abbreviations

DES: Department of Education and Skills
EU: European Union
OECD: Organisation for Economic Co-operation and Development
SFI: Science Foundation Ireland
STEM: Science Technology Engineering Mathematics
UNESCO: United Nations Educational Scientific and Cultural Organization
